# Construct validity and responsiveness of EQ-5D-3L and EQ VAS in psoriatic arthritis: an evaluation based on the Swedish Rheumatology Quality Register

**DOI:** 10.1007/s11136-026-04323-8

**Published:** 2026-07-23

**Authors:** Kinza Degerlund-Maldi, Malin Regardt, Camilla Nystrand Länsman, Lena Larsson, Ioannis Parodis, Emelie Heintz

**Affiliations:** 1 Department of Learning, Informatics, Management and Ethics (LIME), Health Economic and Policy Research Group, Karolinska Institutet, 171 77 Stockholm, Sweden; 2 Stockholm Center for Health Economics, Center for Health Economics, Informatics and Health Services Research (CHIS), Stockholm Healthcare Services, 171 77 Stockholm, Sweden; 3 Medical Unit Allied Health Professionals, Karolinska University Hospital, 171 76 Stockholm, Sweden; 4 Division of Occupational Therapy, Department of Neurobiology, Care Sciences and Society, Karolinska Institutet, Fack 23 200, 141 83 Stockholm, Sweden; 5 Institute of Odontology, Sahlgrenska Academy, University of Gothenburg, Box 450, 405 30 Gothenburg, Sweden; 6 Division of Rheumatology, Department of Medicine Solna, Karolinska Institutet, Karolinska University Hospital, Center for Molecular Medicine (CMM), 171 76 Stockholm, Sweden; 7 Department of Rheumatology, Faculty of Medicine and Health, Örebro University, 701 82 Örebro, Sweden

**Keywords:** Health economics, Patient-reported outcome measures, Quality of life, Psychometric properties, Rheumatology, EQ-5D

## Abstract

**Purpose:**

To evaluate construct validity and responsiveness of the health-related quality of life (HRQoL) instrument EQ-5D-3L (index, dimensions) and EQ VAS among patients with psoriatic arthritis (PsA).

**Methods:**

This retrospective, register-based study utilised data from the Swedish Rheumatology Quality Register. Known-groups validity was assessed by comparing EQ-5D-3L and EQ VAS results across groups with varying levels of physical function or disease activity. Convergent validity was assessed through correlations with comparator instruments. Responsiveness was assessed by analysing correlations between changes in EQ-5D-3L, EQ VAS, and comparator instruments, as well as by assessing the ability to discriminate between patients who improved and those who did not, using the area under the receiver operating characteristic curve (AUC). To confirm construct validity or responsiveness, ≥ 75% of hypotheses had to be supported.

**Results:**

The study included 13,105 patients with PsA. EQ-5D-3L and EQ VAS demonstrated moderate to strong correlations with comparator instruments and found expected differences between groups with varying physical function or disease activity. Over 75% of the hypotheses related to construct validity were supported. Regarding responsiveness, several hypotheses for the EQ-5D-3L were not supported, and none of the AUC-related hypotheses for EQ VAS were supported. Overall, less than 75% of the hypotheses related to responsiveness for EQ-5D-3L and EQ VAS were supported.

**Conclusion:**

The results from this observational study support construct validity of EQ-5D-3L and EQ VAS among patients with PsA. However, responsiveness was not supported for the EQ-5D-3L dimensions which suggests that the EQ-5D-3L may not fully capture changes in HRQoL from interventions impacting other dimensions than pain/discomfort.

**Supplementary Information:**

The online version contains supplementary material available at https://doi.org/10.1007/s11136-026-04323-8.

## Introduction

Health-related quality of life (HRQoL) is a recommended outcome in randomised controlled trials (RCTs) and longitudinal studies involving patients with psoriatic arthritis (PsA) [1]. PsA is a chronic, inflammatory disease, primarily affecting the musculoskeletal system, with common manifestations including spinal, sacroiliac, and peripheral joint inflammation, often leading to joint erosion [2]. PsA can impact physical, emotional, and social aspects of life, and is linked to fatigue, anxiety, and depression [3]. Pain and fatigue are key contributors to reduced HRQoL [3].

Several instruments that measure HRQoL in patients with PsA are available [2]. To ensure that these instruments can capture important differences in HRQoL between groups and changes over time, it is essential to know whether the instruments have good psychometric properties in the intended population [4]. Two critical measurement properties are construct validity, which reflects how well the instrument measures its intended constructs, and responsiveness, which reflects the instrument’s ability to detect changes over time [4]. Instruments lacking these properties may misrepresent HRQoL or fail to capture important changes. If the instrument cannot capture changes, it might give an inaccurate assessment of HRQoL, which can lead to incorrect conclusions about the effects of a treatment.

In patients with PsA, the generic preference-based HRQoL instrument EQ-5D has been used in several RCTs to assess the effect of medication [5, 6], in economic evaluations of treatments [7, 8], and to analyse and compare patient health [9, 10]. The EQ-5D consists of two parts: a questionnaire with five items, each representing a dimension of health (mobility, self-care, usual activities, anxiety/depression, and pain/discomfort), and a visual analogue scale (EQ VAS). Originally developed for use in health economic evaluations, the five items included in the first part of the questionnaire (the descriptive system) can be converted into an index value, which represents the general population’s valuation of the health state [11, 12]. A key advantage of a generic instrument is that it allows comparison across populations and benchmarking against general population norms [13].

EQ-5D has been increasingly incorporated as a patient-reported outcome measure (PROM) in clinical practice [14, 15]. For the purpose of shared decision-making and follow-up of individual patients’ health, it is recommended to focus on the specific dimensions of EQ-5D (mobility, self-care, etc.) rather than the preference-based overall index, which adds a layer of valuations of the general population [16, 17]. During healthcare follow-up, the type and frequency of reported problems are often most relevant to patients and clinicians, as these provide an overall picture of the patients’ problems and can identify the need for support in specific dimensions and guide treatment decisions, something that can be missed when focus is placed on the index value [16].

In Sweden, HRQoL data for patients with PsA are on a national level collected in conjunction with routine PsA follow-up visits using the three-level version of the EQ-5D (EQ-5D-3L) [14]. The collected data, available to patients and healthcare professionals, can be used for shared decision-making and can also be transferred to the Swedish Rheumatology Quality Register (SRQ) [14]. SRQ has been collecting EQ-5D-3L data since 2008 [14], and the registry contains over 500 000 measures with EQ-5D-3L. Since this data is used for research [18] and during patient follow-up, it is important to know how well the EQ-5D-3L can capture HRQoL and changes therein and to know the limitation with the data.

Measurement properties may vary across populations, so assessing construct validity and responsiveness in different populations is essential [19]. A systematic review from 2018 has summarised the literature on the measurement properties of EQ-5D-3L among patients with PsA [20]. The review identified two small cross-sectional studies of fair methodological quality evaluating the construct validity of the EQ-5D-3L index in this patient group [20–22]. Both studies found support for construct validity [21, 22]. Nevertheless, the studies were small (n = 86 and 183) and only one of them [22] compared the results from the EQ-5D-3L with disease-specific instruments. Additionally, none of the studies included in the systematic review evaluated the construct validity of the individual dimensions in the descriptive system of the EQ-5D-3L, nor the EQ VAS. To our knowledge, there have been no studies on the construct validity and responsiveness of EQ-5D-3L among patients with PsA published after the review. Thus, further studies are needed to evaluate the construct validity of the EQ-5D-3L descriptive system and the EQ VAS, as well as the responsiveness of the EQ-5D-3L (index and descriptive system) and the EQ VAS. To address these gaps, this study aimed to evaluate the construct validity and responsiveness of EQ-5D-3L among patients with PsA, in a sample from clinical settings in Sweden.

## Methods

### Study design

This retrospective register-based study followed COSMIN guidelines [4, 23–25]. Construct validity was assessed by comparing the results of EQ-5D-3L and EQ VAS with the results of comparator instruments (convergent validity) and between subgroups (known-groups validity). Responsiveness was assessed by comparing the results of EQ-5D-3L and EQ VAS with the results of comparator instruments. The comparator instruments measure similar and related constructs. Disease activity was measured with Disease Activity Score 28 CRP (DAS28 CRP) or Disease Activity in Psoriatic Arthritis (DAPSA), physical function with Health Assessment Questionnaire Disability Index (HAQ-DI), and pain, fatigue, and general health with three visual analogue scales (VAS). The analyses were based on a priori determined hypotheses formulated based on literature, theory, and discussions with patient research partners, as well as with clinical and methodological experts. To support construct validity or responsiveness, at least 75% of the hypotheses were required to be supported by the data [4, 25]. A study protocol was published (ClinicalTrials.gov ID NCT06568029), and the study was approved by the Swedish Ethical Review Authority (registration number: 2023-04394-01).

### Study population and data

Data on disease activity, physical function, fatigue, pain, general health, and HRQoL (measured with EQ-5D-3L) linked to healthcare visits were extracted from the SRQ. Patients completed PROMs via a digital platform in connection with their healthcare visit at a rheumatology clinic, and healthcare providers contributed with clinical data. The data was subsequently transferred to the SRQ, if the patients did not opt-out. For this study, we included all healthcare visits of patients ≥ 18 years with a diagnosis of PsA in the SRQ. The participants also had to have a complete registration of responses in EQ-5D-3L and at least one measurement with DAS28 or DAPSA in conjunction with the same visit as EQ-5D-3L. For construct validity, the most recent complete registration was used, if patients had more than one. For responsiveness, the first two complete registrations within the first year after diagnosis were selected. The data in the study was collected between 2007 and 2024 (see Table 1). In total, 55% of the sample consisted of patients from the three largest regions in Sweden (Region Stockholm, Västra Götaland, and Skåne), whereas the remaining regions each accounted for between 0.1 and 5.2% of the sample.

**Table 1:** Background characteristics

	Newly diagnosed		Total population n = 13,105
	visit 1 n = 2,264	visit 2 n = 2,264	
Female (%)	51.2		50.9
Age at diagnosis, mean (SD)	50.04 (15.10) ^a^		46.68 (14.59) ^b^
Age at EQ-5D-3L registration, mean (SD)	50.7 (14.53)	51.10 (14.52)	56.31 (13.98)
Days between visit 1 and 2 (SD)	142.57 (74.86)		
DAPSA, mean (SD)	21.33 (12.12)	14.40 (10.81)	12.24 (9.95)
DAS28 CRP, mean (SD)	3.65 (1.19)	2.98 (1.16)	2.71 (1.10)
HAQ-DI, mean (SD)	0.82 (0.56)	0.67 (0.56)	0.68 (0.60)
Pain, mean (SD)	49.62 (24.73)	38.23 (26.32)	38.06 (27.00)
Fatigue, mean (SD)	50.37 (28.11)	42.71 (28.94)	41.95 (29.26)
General health, mean (SD)	47.83 (25.04)	38.28 (26.11)	38.24 (26.75)
EQ-5D-3L index, mean (SD)	0.526 (0.315)	0.614 (0.300)	0.625 (0.305)
EQ VAS, mean (SD)	60.33 (21.89)	65.46 (21.27)	65.69 (21.64)
EQ-5D-3L dimensions			
Mobility			
No problems	40.9%	51.8%	54.1%
Some problems	59.1%	48.0%	45.7%
Unable	0.1%	0.2%	0.3%
Self-care			
No problems	79.1%	84.0%	84.2%
Some problems	20.2%	15.4%	15.0%
Unable	0.7%	0.6%	0.8%
Usual activities			
No problems	48.4%	60.1%	64.6%
Some problems	44.9%	35.6%	31.6%
Unable	6.7%	4.3%	3.9%
Pain/discomfort			
No problems	4.0%	10.8%	14.4%
Moderate problems	72.1%	74.2%	70.2%
Extreme problems	23.9%	15.0%	15.4%
Anxiety/depression			
No problems	43.8%	51.8%	55.8%
Moderate problems	49.4%	42.1%	38.4%
Extreme problems	6.8%	6.1%	5.8%
Full health	3.0%	9.0%	11.4%
Year of the visit	Number of patients having a visit each year		
2007	0	0	1
2008	0	0	1
2009	116	57	38
2010	218	191	100
2011	139	173	171
2012	154	147	244
2013	178	159	389
2014	188	198	544
2015	185	177	461
2016	160	174	533
2017	156	153	693
2018	178	162	997
2019	159	182	1373
2020	96	121	882
2021	111	89	1071
2022	83	104	1551
2023	110	92	2044
2024	33	85	2012

DAPSA, Disease Activity Index in Psoriatic Arthritis; DAS28 CRP, Disease Activity Score 28 CRP; HAQ-DI, Health Assessment Questionnaire Disability Index; SD, Standard deviation

^a^ 42% had missing diagnosis date, ^b^ 39% had missing diagnosis date

#### Health-related quality of life

EQ-5D-3L includes a descriptive system with five items, each representing a dimension of health: mobility, self-care, usual activities, pain/discomfort, and anxiety/depression, and EQ VAS [11, 12]. In this study, the Swedish version of the EQ-5D-3L was used, in which the wording of the anxiety/depression dimension differs slightly from the English version (translated as worry/low mood). Each item has three levels: no problems (level 1), some problems (level 2), and extreme problems (level 3). Responses across the five dimensions can be combined to generate a five-digit code representing a specific health state. In total, the responses can be combined into 243 unique health states. These health states can be translated into a single index using country-specific value sets. An EQ-5D-3L index of 1 represents the value of full health, and 0 represents a value equal to being dead. In this study, the primary analysis employed the EQ-5D-3L UK value set [26], while a Swedish experience-based value set was used for a sensitivity analysis [27]. EQ VAS captures the respondent’s self-rated health from “the best health you can imagine” to “the worst health you can imagine” [11, 12].

#### Disease activity

DAS28 contains questions about swollen and tender joints, disease activity, and inflammation [28]. DAPSA contains questions about swollen and tender joints, disease activity, pain, and inflammation [29]. There are several studies supporting the construct validity and responsiveness of DAS28 and DAPSA in patients with PsA [29–35]. DAS28 CRP, which has been used in this study, ranges between 0 (remission, no disease activity) and 9.4 (highest possible disease activity) [36]. Less than 4 in DAPSA indicates remission, and greater than 28 indicates high disease activity [37].

#### Physical function

HAQ-DI measures physical function through questions about mobility, daily activities, and self-care [38]. Several studies support the construct validity and responsiveness of HAQ-DI in patients with PsA [20, 39]. HAQ-DI ranges from 0 (higher physical function) to 3 (lower physical function) [38].

#### Visual analogue scale measuring pain, fatigue, and general health

The SRQ contains three questions about pain, fatigue, and general health, all measured with a VAS [40]; see supplementary material for the three VAS. The questions ask how much pain the person has had, how fatigued they have been, and how their general health has been in the last week due to PsA. The scale ranges from no problem (0) to as bad as it can be (100) [40]. A systematic review found limited support for construct validity and responsiveness for VAS pain and general health, and a lack of studies assessing VAS fatigue in patients with PsA [20]. Nevertheless, these questions were included in the analyses as pain and fatigue are commonly reported problems in patients with PsA, and there is support for construct validity and responsiveness of all three VAS in patients with rheumatoid arthritis (RA) [41–45].

### Analyses

Data were analysed using IBM SPSS Statistics (Version 29). Analyses were conducted using available cases.

#### Construct validity

The analyses of convergent validity involved testing a priori hypotheses regarding the correlation of results from the EQ-5D-3L and EQ VAS with comparator instruments measuring *similar* or *related* constructs; see supplementary file table S1 for the hypothesised correlations [4, 23, 24]. Related constructs were expected to have at least a moderate correlation (≥ 0.3), while similar constructs were expected to have a strong correlation (≥ 0.5) [23]. Due to tied ranks, Kendall’s Tau was used for EQ-5D-3L dimensions, and Spearman’s for EQ-5D-3L index and EQ VAS.

Known-groups validity was assessed by testing a priori hypotheses about expected differences in the EQ-5D-3L index, the EQ-5D-3L dimensions, and the EQ VAS between patient groups based on disease activity or physical function [24]. Patients with lower disease activity or higher physical function (< 3.2 DAS28 CRP, ≤ 14 DAPSA, < 1 HAQ-DI) were expected to report less problems in the EQ-5D-3L dimensions mobility, self-care, usual activities, and pain/discomfort and to have a higher EQ-5D-3L index and a higher EQ VAS compared to patients with higher disease activity or lower physical function (≥ 3.2 DAS28, > 14 DAPSA, ≥ 1 HAQ-DI). The thresholds for levels of disease activity and physical function were based on the literature [46–49]. Mann–Whitney U tested differences in the distribution of responses in the descriptive system. To define the size of the differences in reported problems in the EQ-5D-3L dimensions, effect sizes (r) were calculated. An effect size of ≥ 0.1 was considered small, ≥ 0.3 medium, and ≥ 0.5 large [50, 51]. To support the hypotheses regarding the dimensions, we expected at least a small effect size (≥ 0.1). For the EQ-5D-3L index and the EQ VAS, differences between groups were interpreted using minimally important differences (MID). A MID of 0.05 was applied for the EQ-5D-3L index, based on individuals with RA [52], and a MID of 10.3 was used for the EQ VAS, derived from individuals with musculoskeletal conditions [53].

#### Responsiveness

Responsiveness was assessed by analysing the correlation of changes in the EQ-5D-3L and EQ VAS with changes in the comparator instruments [4, 23, 24]. Hypotheses mirrored those for convergent validity. Furthermore, to assess the ability of the EQ-5D-3L index and the EQ VAS to differentiate between patients who improved and those who did not, the area under the receiver operating characteristic curve (AUC) was calculated [4, 23, 24]. Patients were considered to have improved based on response criteria from the literature. The response criteria for DAPSA were ≥ 50% improvement [37]. The response criteria for DAS28 CRP were > 1.2 if DAS28 CRP was > 5.1 at visit two and > 0.6 if DAS28 CRP was ≤ 5.1 at visit two [36]. The response criteria for HAQ-DI were ≥ 0.22 points improvement on the scale [54]. We hypothesised an AUC ≥ 0.70 [4, 24]. For responsiveness, the first two complete registrations within the first year after diagnosis were selected, reflecting the likelihood of changes in health status during this period. When the date of diagnosis was unavailable, the date of inclusion in the SRQ was used as a proxy. Additionally, a sensitivity analysis included only patients with a diagnosis date ≤ 365 days.

## Results

### Construct validity: EQ-5D-3L

**Fig. 1:**
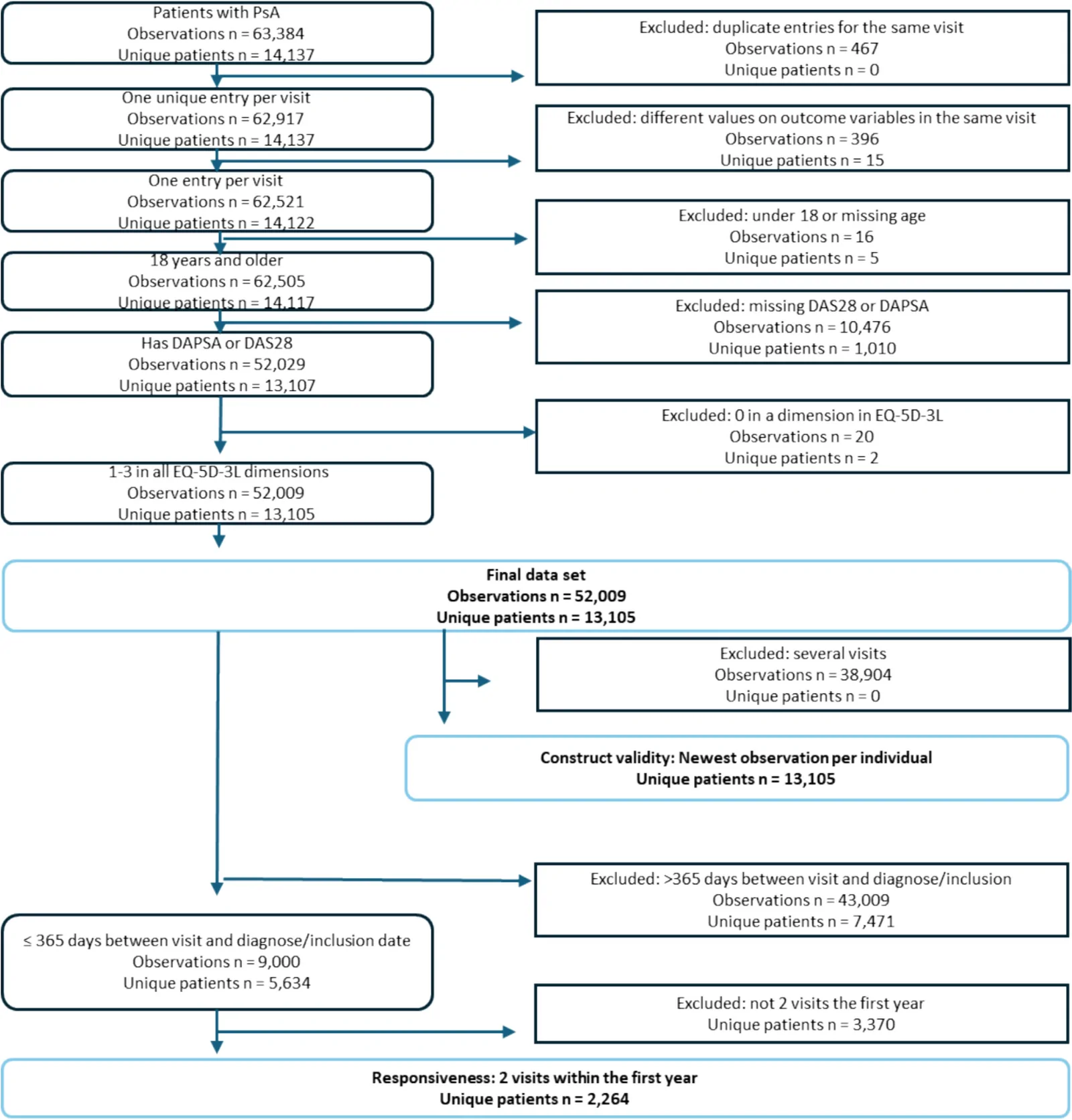
Flowchart describing the population included in the study. DAPSA, Disease Activity Index in Psoriatic Arthritis; DAS28 CRP, Disease Activity Score 28 CRP, PsA, Psoriatic Arthritis

For the analysis of construct validity, the last complete measure was used, which included 13,105 measurements and patients (Fig. 1). Of the population, 50.9% were female, with a mean age of 56.3 (SD 14.0) years. On average, patients had a DAPSA score of 12.24 (SD 9.95) and a DAS28 CRP score of 2.71 (SD 1.10), indicating low disease activity.

The mean EQ-5D-3L index was 0.63 (SD 0.31), and 11.4% of patients reported full health (Table 1). The EQ-5D-3L item in which problems were most frequently reported was pain/discomfort, where only 14.4% reported no problems.

#### Convergent validity

The EQ-5D-3L index correlated strongly with all comparator instruments, and all hypotheses were supported (≥ 0.5, see Table 2). For the descriptive system, 17 of 20 correlations were in line with our hypotheses, moderate or strong. The three hypotheses not supported by the data were related to the EQ-5D-3L dimension self-care, where the correlations with the comparator instruments (DAS28 CRP, DAPSA, and VAS fatigue) were below 0.3.

**Table 2:**
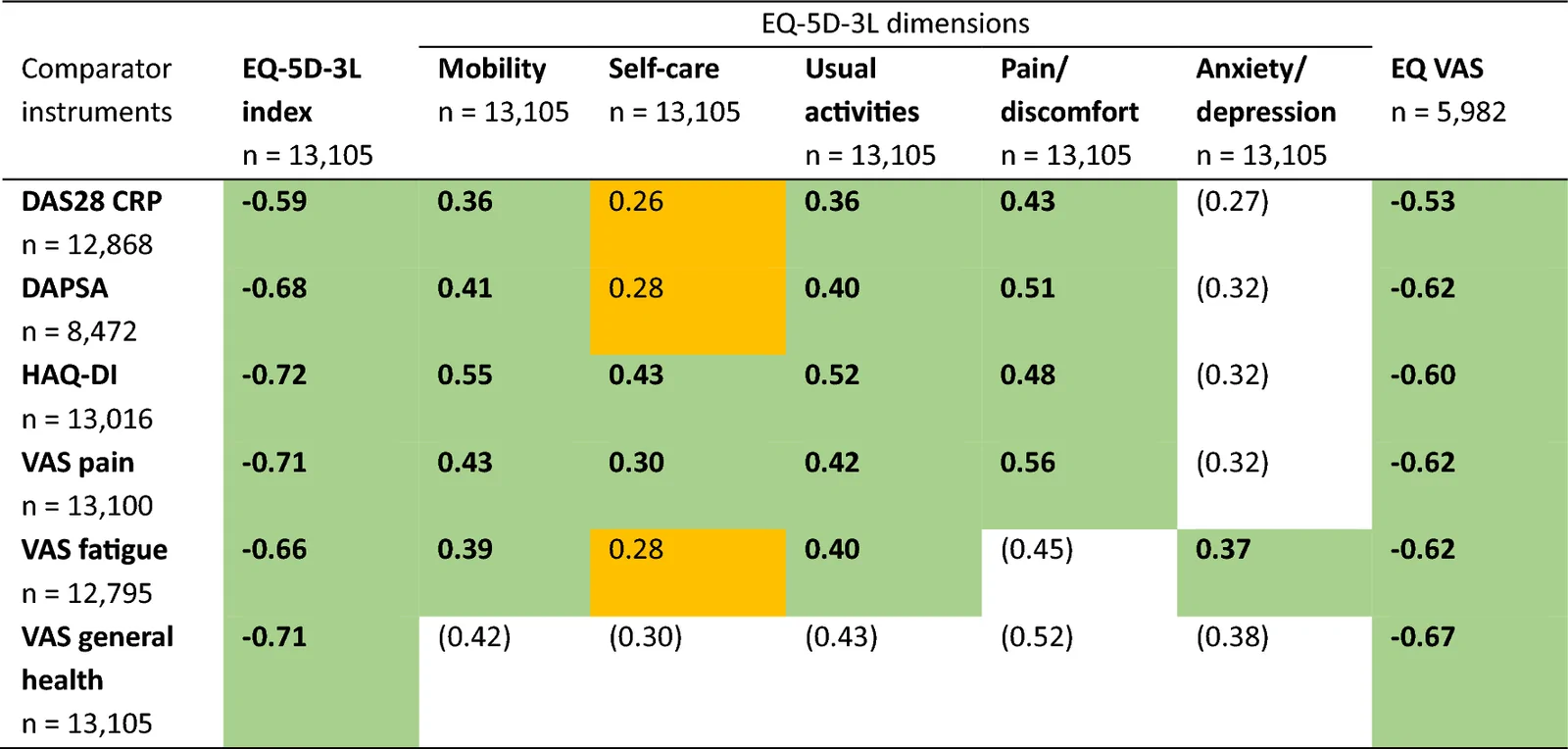
Convergent validity: correlations between EQ-5D-3L (index and descriptive system), EQ VAS, and comparator instruments

Bold text with green shading indicates that the hypothesis was supported, while regular text with yellow shading indicates that the hypothesis was not supported. Correlations shown in brackets represent cases where no hypothesis was specified. Spearman’s rho was used for the EQ-5D-3L index and EQ VAS, and Kendall’s tau was used for the descriptive system. All correlations are significant at the 0.01 level (two-tailed)

DAPSA, Disease Activity Index in Psoriatic Arthritis; DAS28 CRP, Disease Activity Score 28 CRP; HAQ-DI, Health Assessment Questionnaire Disability Index; VAS, Visual Analogue Scale

#### Known-groups validity

The differences in mean EQ-5D-3L index between patients with higher versus lower disease activity or physical function measured with DAS28 CRP, DAPSA, and HAQ-DI were 0.28, 0.33, and 0.37, respectively (Table 3), which was substantially higher than the MID of 0.05. In each of the five dimensions, patients with either higher disease activity (measured with DAS28 CRP or DAPSA) or lower physical function (measured with HAQ-DI) reported statistically significantly more problems compared to patients with either lower disease activity or higher physical function. All the effect sizes were above the small (≥ 0.1) hypothesised effect size. Thus, all hypotheses for known-groups validity were supported by the data.

**Table 3:**
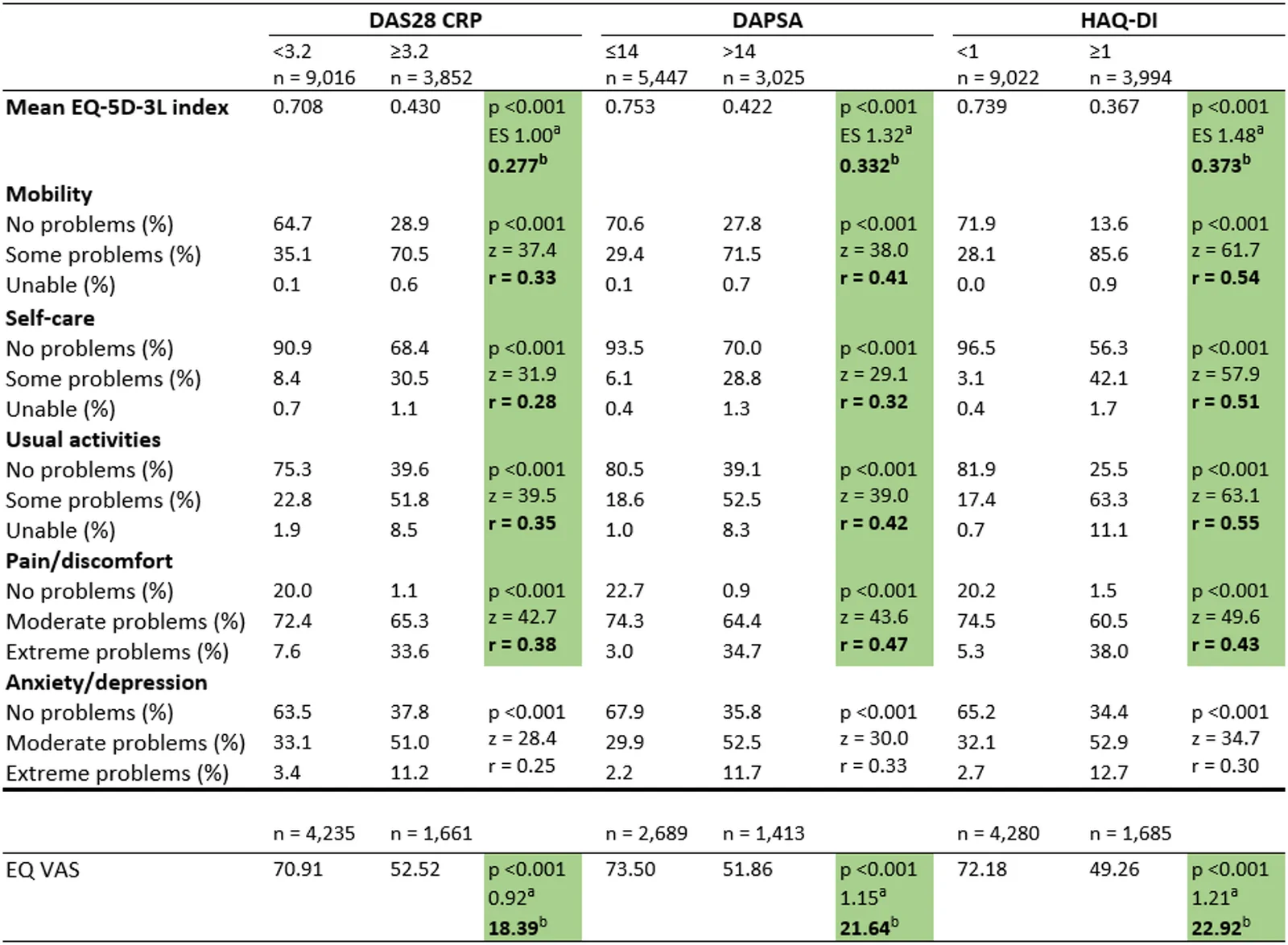
Known-groups validity: mean EQ-5D-3L index, EQ VAS, and responses to the descriptive system

Bolded effect sizes or differences in the EQ-5D-3L index or EQ VAS, along with green shading, indicate that the hypothesis was supported. No hypotheses were specified for the anxiety/depression item.DAPSA, Disease Activity Index in Psoriatic Arthritis; DAS28 CRP, Disease Activity Score 28 CRP; ES, effect size; HAQ-DI, Health Assessment Questionnaire Disability Index; p, p-value; r, effect size; VAS, Visual Analogue Scale; Z, Z-score Mann-Whitney U test. a Effect size was calculated using Cohen’s d, b Difference in mean EQ-5D-3L index /EQ VAS between the group

In total, 38 of 41 (93%) hypotheses for construct validity of the EQ-5D-3L were supported.

Sensitivity analyses using the Swedish experience-based EQ-5D-3L value set yielded similar results (see supplementary material table S2-S4).

### Responsiveness: EQ-5D-3L

Of the total sample, 2,264 patients had their diagnosis for ≤ 365 days at the time of their first two complete visits. The time between the first and second visit was on average 142.57 (SD 74.86) days. At the first visit, patients had an average DAPSA score of 21.33 (SD 12.12) and an average DAS28 score of 3.65 (SD 1.19) indicating moderate disease activity. The mean EQ-5D-3L index increased from 0.53 (SD 0.32) to 0.61 (SD 0.30) between the visits, and the proportion of patients who reported full health increased from 3.0% to 9.0%. The most frequently reported problem was in the EQ-5D-3L dimension pain/discomfort, where only 4.0% reported no problem at the first visit and 10.8% at the second visit. Ceiling effects were observed in the items for the dimensions mobility, self-care, usual activities, and anxiety/depression with 40.9%, 79.1%, 48.4%, and 43.8% of patients, respectively, reporting no problem at the first visit (Table 1). See figure S1 in supplementary material for Sankey diagrams illustrating the transition of patient responses across the EQ-5D-3L descriptive system for patients who improved and did not improve based on DAS28 CRP.

The correlations between the changes in the EQ-5D-3L index and the changes in the comparator instruments were moderate to strong, all in line with the hypothesised thresholds (Table 4). In contrast, none of the hypotheses regarding changes in the EQ-5D-3L item for self-care and changes in the comparator instruments were supported by the data, as all correlations were below 0.30 (ranging from 0.12 to 0.25). Further, the correlations between changes in the EQ-5D-3L items for mobility and usual activities and the comparator instruments were lower than expected in 3/5 and 4/5 of the analyses, respectively. The correlations between changes in the EQ-5D-3L dimension pain/discomfort did correlate as expected with the changes in the comparator instruments in most (3/4) of the analyses. However, it should be noted that the correlation between the change in this dimension and the change in VAS pain was only moderate (0.39), which was lower than expected.

**Table 4:**
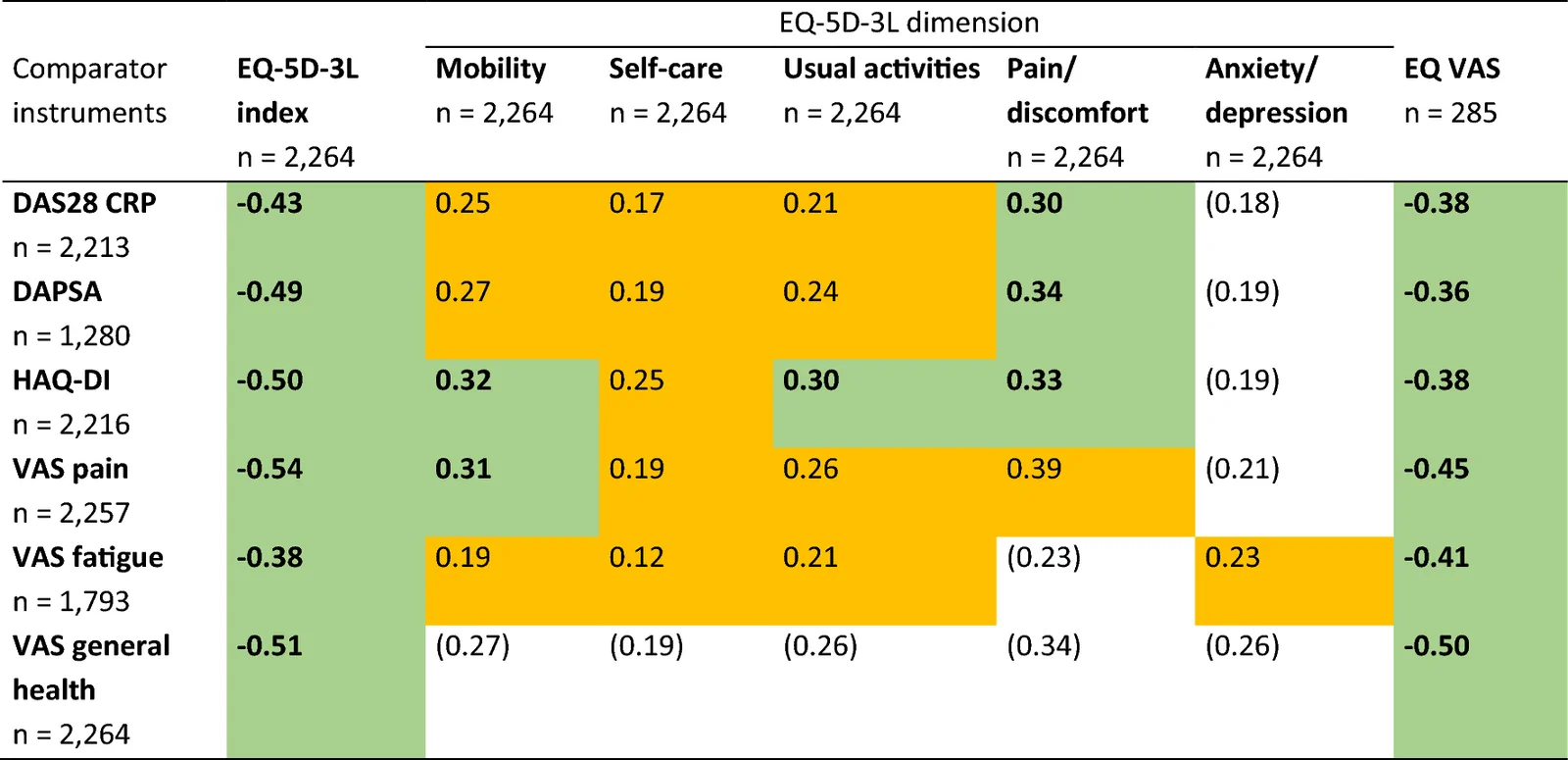
Responsiveness: correlations between changes in EQ-5D-3L (index and descriptive system), EQ VAS, and comparator instruments

Bold text with green shading indicates that the hypothesis was supported, while regular text with yellow shading indicates that the hypothesis was not supported. Correlations shown in brackets represent cases where no hypothesis was specified. Spearman’s rho was used for the EQ-5D-3L index and EQ VAS, and Kendall’s tau was used for the descriptive system. All correlations are significant at the 0.01 level (two-tailed). DAPSA, Disease Activity Index in Psoriatic Arthritis; DAS28 CRP, Disease Activity Score 28 CRP; HAQ-DI, Health Assessment Questionnaire Disability Index; VAS, Visual Analogue Scale

Based on DAS28 CRP, DAPSA, and HAQ-DI, improvement was observed in 1061 (47.9%), 471 (36.8%), and 882 (39.8%) patients, respectively. The AUCs exceeded 0.7 in all three analyses (Table 5), indicating that the EQ-5D-3L index was able to discriminate between patients who had improved and those who had not improved.

**Table 5:**
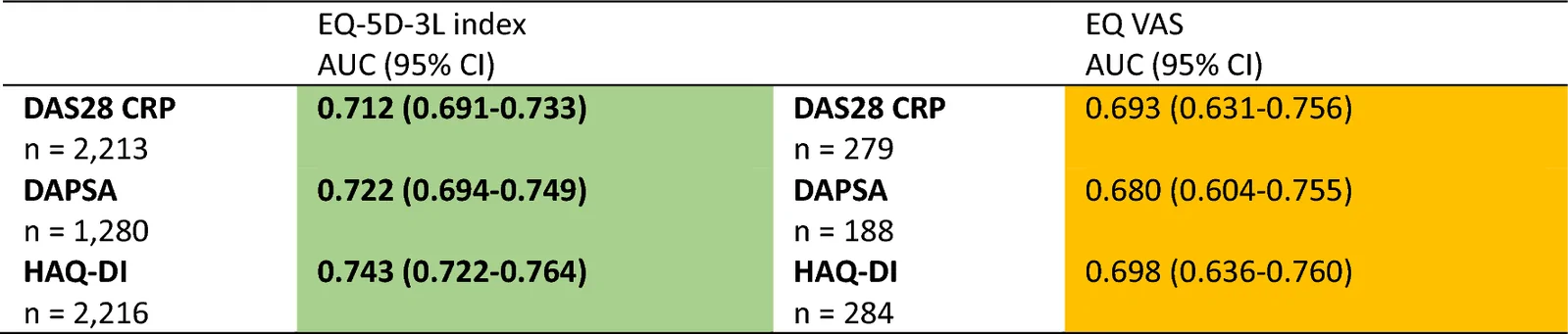
Area under the receiver operating characteristic curve for the EQ-5D-3L index and EQ VAS

Bold text with green shading indicates that the hypothesis was supported, while regular text with yellow shading indicates that the hypothesis was not supported. AUC, Area Under the Curve; CI, Confidence Interval; DAPSA, Disease Activity Index in Psoriatic Arthritis; DAS28 CRP, Disease Activity Score 28 CRP; HAQ-DI, Health Assessment Questionnaire Disability Index

In total, 15 of 29 (52%) hypotheses for responsiveness of the EQ-5D-3L (index and descriptive system) were supported.

The sensitivity analysis, in which we only included patients with a date of diagnosis (see supplementary material table S5), did not change the results in the correlation-based approach. However, the AUC decreased from 0.71 to just below 0.7 (0.695) based on DAS28 CRP (see supplementary material table S6). Sensitivity analyses using the Swedish experience-based EQ-5D-3L value set yielded similar results as the main analysis (see supplementary material table S2).

### Construct validity: EQ VAS

In the analysis for EQ VAS, a total of 5,982 patients and measurements were included. All correlations between the EQ VAS and the comparator instruments were strong (≥ 0.5) and in line with the hypothesised thresholds (Table 2). For the total sample, the mean EQ VAS was 65.69 (SD 21.64). The mean difference in EQ VAS between patients with high versus low disease activity or physical function, measured with DAS28 CRP, DAPSA, and HAQ-DI, was 18.4, 21.6, and 22.9, respectively (Table 3). These differences were higher than the MID of 10.3. Thus, 9 of 9 hypotheses for the construct validity of EQ VAS were supported.

### Responsiveness: EQ VAS

In the analyses of the responsiveness of EQ VAS, 285 patients were included. The mean EQ VAS increased from 60.33 (SD 21.89) to 65.46 (SD 21.27) between the first and second visit (Table 1). All correlations between changes in EQ VAS and changes in the comparator instruments were moderate to strong and in line with the hypotheses (Table 4). Based on DAS28 CRP, DAPSA, and HAQ-DI, improvement was seen in 146 (52.3%), 83 (44.1%), and 116 (40.8%) patients, respectively, with AUCs values just below 0.7 in all analyses (Table 5).

In total, 6 of 9 (67%) hypotheses for responsiveness of EQ VAS were supported.

The results in the correlation-based approach did not change in the sensitivity analysis when we only included patients with a date of diagnosis (see supplementary material table S5). However, the AUC increased to 0.71 based on both DAS28 CRP and HAQ-DI (see supplementary material table S6).

## Discussion

Using data from a large Swedish clinical population, we found support for the construct validity of EQ-5D-3L and EQ VAS in patients with PsA. Of the a priori hypotheses, 93% were confirmed for the EQ-5D-3L (index and descriptive system) and all for the EQ VAS, indicating their suitability for measuring HRQoL cross-sectionally among patients with PsA. EQ-5D-3L can, for example, be used to compare HRQoL across patients with different levels of disease activity and to assess the type of problems reported within the patient group. Our study is, to our knowledge, the first study to assess the responsiveness of the EQ-5D-3L and EQ VAS in this patient group. Responsiveness was not supported as only 52% of the hypotheses were supported by the data for the EQ-5D-3L and 67% for the EQ VAS.

Our results regarding construct validity of the EQ-5D-3L index are supported by a systematic review [20], which found two studies evaluating construct validity of the EQ-5D-3L index, with results similar to our study [21, 22]. Both Brodszky et al. and Leung et al. found that the EQ-5D-3L index could discriminate between groups and correlated as expected with selected comparator instruments [21, 22]. Our results are also supported by an analysis of patients with ankylosing spondylitis (AS), which was based on the same method as the current study, also used data from the SRQ, and showed similar results regarding construct validity [55].

For the responsiveness analysis of the EQ VAS, all hypotheses regarding correlations between changes in EQ VAS and changes in the comparator instruments were supported. However, AUC values fell just below the threshold in all three primary analyses, while two of the three sensitivity analyses (restricted to patients with a recorded diagnosis date) produced values just above the threshold. Overall, these findings highlight the need for further research.

Notably, all hypotheses concerning the responsiveness of the EQ-5D-3L index were supported, indicating its ability to detect changes over time. However, the responsiveness of the dimensions was generally not supported, as only the responsiveness of the dimension for pain/discomfort was supported. This means that when using EQ-5D-3L to measure changes over time, changes in the other dimensions might be underestimated. Consequently, even though it has been recommended to use the results from the individual dimensions when EQ-5D is used in clinical practice [16], our findings suggest that one should be cautious when using the results from the EQ-5D-3L dimensions mobility, self-care, usual activities, and anxiety/depression over time in this patient group. The poor responsiveness of the EQ-5D-3L dimensions also raises concerns about the responsiveness of the EQ-5D-3L index in contexts where changes are expected in dimensions other than pain/discomfort. The EQ-5D-5L, which has five response levels instead of three, was developed to improve responsiveness and sensitivity [56]. It also incorporates revised wording, for example, in the mobility dimension “confined to bed” was changed to “unable to walk about”, which may further enhance responsiveness. Thus, the EQ-5D-5L could be an alternative instrument for patients with PsA, and future studies of the construct validity and responsiveness of the descriptive system of EQ-5D-5L are recommended. Additionally, the EuroQol is developing dimensions that can be added to the EQ-5D instrument, known as bolt-ons [57]. While keeping the instrument brief, bolt-ons aim to improve the measurement properties of the instrument. There is ongoing work developing a bolt-on for tiredness [58], and as fatigue is a central symptom in PsA, this bolt-on could be relevant for this patient group.

A possible explanation for the relatively strong performance of the pain/discomfort dimension is the absence of ceiling effects, as only 4% of patients reported no problems in this item at the first visit, compared to 41%, 80%, 48%, and 44% for mobility, self-care, usual activities, and anxiety/depression, respectively. The high ceiling effects in the other four dimensions may have contributed to the low correlations for responsiveness, as many patients could not report improvement in the EQ-5D-3L items despite reporting improvements in the comparator instruments. Ceiling effects in the items of the EQ-5D-3L descriptive system have been reported in patients with PsA [9, 10] and in other populations in Sweden [59]. It is noteworthy that the hypothesised correlation between changes in VAS pain and changes in the EQ-5D-3L pain/discomfort item was below the hypothesised strength of  ≥ 0.5, suggesting limited ability to fully detect changes in pain over time. While limited responsiveness of the VAS for pain itself in patients with PsA could be a contributing factor [20], previous studies in related populations, such as RA, support the responsiveness of VAS pain [43–45].

One of the key strengths of this study is the adherence to the COSMIN guidelines, ensuring a systematic and methodologically sound evaluation process [4, 23–25]. We employed established comparator instruments, DAS28 CRP, DAPSA, and HAQ-DI, that have demonstrated construct validity and responsiveness, enhancing the credibility of our findings. The formulation of hypotheses was carried out in collaboration with patient research partners and clinical and methodological experts. Additionally, we applied both a UK [26] and a Swedish [27] EQ-5D-3L value set and sensitivity analyses confirmed that our findings remained consistent regardless of the value set used. Moreover, data from a large nationwide registry covering routine care between the years 2007 and 2024 reduces selection bias and supports the generalisability of the findings, particularly for settings where the Swedish version of the EQ-5D-3L is used. However, because patients in our sample on average had low to moderate disease activity, caution is warranted when generalising these results to populations with substantially different disease profiles.

### Limitations

The data is observational registry data, and we included patients who had responses to the EQ-5D-3L descriptive system or EQ VAS. However, we, do not have information about the patients who did not complete the EQ-5D-3L, nor do we have information about the patients who opted out of SRQ. This could potentially introduce selection bias. Nonetheless, the coverage rate for PsA in the registry is 80.5% [60]. Moreover, the population included patients with different disease activity and physical function, even though the average disease activity was low (construct validity) and moderate (responsiveness). This can affect generalisability to patients with substantially different disease profiles. On the other hand, by using registry data we capture the patients that will use EQ-5D-3L in a clinical setting. Further, we used EQ-5D-3L data and data from the comparator instrument connected to the same visit. However, the measurement conditions could have changed if the instruments were filled out on different days. Nonetheless, given the chronic nature of PsA, we judge this to be unlikely. The responsiveness analyses included patients with PsA who had received their diagnosis within one year at both the first and second visits. For those without a recorded diagnosis date, selection was instead based on the date of registration in the SRQ. These patients may have had longer disease duration, and sensitivity analyses showed some differing AUC results for EQ VAS. In evaluating known-groups validity and responsiveness, data on disease activity and physical function were used to categorise patients. However, patients may also have differed in other relevant characteristics, such as the prevalence of comorbidities. As comorbidity data were not available, we were unable to adjust for it, and it is therefore possible that differences in the EQ-5D-3L and the EQ VAS reflected not only disease activity and physical function related to PsA, but also the presence of additional health issues. We recommend future studies to adjust for comorbidities. Finally, we used three VAS that measure pain, fatigue, and general health. Although support for their construct validity and responsiveness among patients with PsA is limited, there is support for construct validity and responsiveness in patients with RA [41–45].

## Conclusion

The findings support the construct validity of the EQ-5D-3L and EQ VAS in assessing HRQoL among patients with PsA, indicating that the EQ-5D-3L and EQ VAS can be used for cross-sectional measurement in this population, for example to compare HRQoL between groups or to study what problems the patient group has. Furthermore, the results suggest that the EQ-5D-3L index is sensitive to changes in HRQoL over time in patients with PsA. However, responsiveness of the EQ-5D-3L dimensions was supported only for the pain/discomfort dimension. Given that the EQ-5D-3L index is derived from all five dimensions, this may limit its ability to fully capture changes in HRQoL resulting from interventions targeting aspects of health beyond pain/discomfort. Caution is warranted when interpreting changes in the EQ-5D-3L index and individual dimensions over time, and the use of the index or dimensions as standalone indicators of change in health status among patients with PsA should be approached with care. As the increased number of response levels in EQ-5D-5L may improve its ability to detect changes over time, future studies are recommended to investigate the construct validity and responsiveness of the EQ-5D-5L in patients with PsA. Additionally, our findings highlight the need for future research of the responsiveness of EQ VAS. Because patients in our sample on average had low to moderate disease activity, some caution is warranted when interpreting these findings and generalising them to populations with substantially different disease profiles.

## Supplementary Information

Below is the link to the electronic supplementary material.

Supplementary file1 (DOCX 181 KB)

## Data Availability

Access to data is restricted by Swedish law. General information about obtaining access to data is available from the corresponding author KDM upon request.

## References

[1] Orbai, A. M., de Wit, M., Mease, P. J., Callis Duffin, K., Elmamoun, M., Tillett, W., & Ogdie, A. (2017). Updating the psoriatic arthritis (PsA) core domain set: a report from the PsA workshop at OMERACT 2016. *Journal of Rheumatology*. 10.3899/jrheum.160904 28148697 PMC5538953

[2] FitzGerald, O., Ogdie, A., Chandran, V., Coates, L. C., Kavanaugh, A., Tillett, W., & Mease, P. J. (2021). Psoriatic arthritis. *Nature Reviews Disease Primers*. 10.1038/s41572-021-00293-y 34385474

[3] James, L., Hailey, L. H., Suribhatla, R., McGagh, D., Amarnani, R., Bundy, C. E., & Coates, L. C. (2024). The impact of psoriatic arthritis on quality of life: a systematic review. *Ther Adv Musculoskelet Dis*. 10.1177/1759720x241295920 39717741 PMC11664531

[4] Mokkink, L. B., Elsman, E. B. M., & Terwee, C. B. (2024). COSMIN guideline for systematic reviews of patient-reported outcome measures version 2.0. *Quality of life research : an international journal of quality of life aspects of treatment, care and rehabilitation,**33*(11), 2929–2939. 10.1007/s11136-024-03761-6 39198348 PMC11541334

[5] Gladman, D. D., Mease, P. J., Gossec, L., Husni, M. E., Gottlieb, A. B., Ink, B., & Tillett, W. (2025). Effect of Bimekizumab on Patient-Reported Outcomes and Work Productivity in Patients With Psoriatic Arthritis: 1-Year Results From 2 Phase III Studies. *The Journal of Rheumatology,**52*(5), 466–478. 10.3899/jrheum.2024-0923 39892885

[6] Lu, Y., Dai, Z., Lu, Y., & Chang, F. (2022). Effects of bDMARDs on quality of life in patients with psoriatic arthritis: Meta-analysis. *British Medical Journal Open,**12*(4), Article e058497. 10.1136/bmjopen-2021-058497 PMC9006847 35414559

[7] Antonazzo, I. C., Gribaudo, G., La Vecchia, A., Ferrara, P., Piraino, A., Cortesi, P. A., & Mantovani, L. G. (2024). Cost and Cost Effectiveness of Treatments for Psoriatic Arthritis: An Updated Systematic Literature Review. *PharmacoEconomics,**42*(12), 1329–1343. 10.1007/s40273-024-01428-1 39182010

[8] D’Angiolella, L. S., Cortesi, P. A., Lafranconi, A., Micale, M., Mangano, S., Cesana, G., & Mantovani, L. G. (2018). Cost and Cost Effectiveness of Treatments for Psoriatic Arthritis: A Systematic Literature Review. *PharmacoEconomics,**36*(5), 567–589. 10.1007/s40273-018-0618-5 29441473

[9] Moraes, F. A., Da Silva, M. R. R., Dos Santos, J. B. R., Acurcio, F. A., Almeida, A. M., Kakehasi, A. M., & Alvares-Teodoro, J. (2021). Health-Related Quality of Life in Psoriatic Arthritis: Findings and Implications. *Value in Health Regional Issues,**26*, 135–141. 10.1016/j.vhri.2021.06.003 34390960

[10] Krüger, K., Burmester, G. R., Wassenberg, S., Bohl-Bühler, M., & Thomas, M. H. (2019). Patient-reported outcomes with golimumab in patients with rheumatoid arthritis, psoriatic arthritis, and ankylosing spondylitis: Non-interventional study GO-NICE in Germany. *Rheumatology International,**39*(1), 131–140. 10.1007/s00296-018-4180-4 30415451 PMC6329737

[11] Brooks, R. (1996). EuroQol: The current state of play. *Health policy (Amsterdam, Netherlands),**37*(1), 53–72. 10.1016/0168-8510(96)00822-6 10158943

[12] EuroQol Group. (1990). EuroQol–a new facility for the measurement of health-related quality of life. *Health Policy (Amsterdam, Netherlands),**16*(3), 199–208. 10.1016/0168-8510(90)90421-9 10109801

[13] Fayers, P. M., & Machin, D. (2016). *Quality of life: The assessment, analysis, and reporting of patient-reported outcomes* (3rd ed.). Wiley Blackwell.

[14] Ernstsson, O., Janssen, M. F., & Heintz, E. (2020). Collection and use of EQ-5D for follow-up, decision-making, and quality improvement in health care - the case of the Swedish National Quality Registries. *Journal of Patient-Reported Outcomes,**4*(1), 78. 10.1186/s41687-020-00231-8 32936347 PMC7494720

[15] Devlin, N. J., & Brooks, R. (2017). EQ-5D and the EuroQol Group: Past, Present and Future. *Applied Health Economics and Health Policy,**15*(2), 127–137. 10.1007/s40258-017-0310-5 28194657 PMC5343080

[16] Appleby, J., Devlin, N., & Parkin, D. (2016). *Using Patient Reported Outcomes to Improve Health Care*. Newark, UNITED KINGDOM: John Wiley & Sons, Incorporated. Retrieved from http://ebookcentral.proquest.com/lib/ki/detail.action?docID=4179324

[17] Devlin, N. J., Parkin, D., & Browne, J. (2010). Patient-reported outcome measures in the NHS: New methods for analysing and reporting EQ-5D data. *Health Economics,**19*(8), 886–905. 10.1002/hec.1608 20623685

[18] Teni, F. S., Rolfson, O., Devlin, N., Parkin, D., Nauclér, E., Burström, K., & Swedish Quality Register (SWEQR) Study Group. (2022). Longitudinal study of patients’ health-related quality of life using EQ-5D-3L in 11 Swedish National Quality Registers. *British Medical Journal Open,**12*(1), Article e048176. 10.1136/bmjopen-2020-048176 PMC8739074 34992101

[19] Streiner, D. L., Norman, G. R., & Cairney, J. (2015). *Health Measurement Scales: A Practical Guide to Their Development and Use*. Oxford, UNITED KINGDOM: Oxford University Press, Incorporated. Retrieved from http://ebookcentral.proquest.com/lib/ki/detail.action?docID=1816173

[20] Højgaard, P., Klokker, L., Orbai, A. M., Holmsted, K., Bartels, E. M., Leung, Y. Y., & Christensen, R. (2018). A systematic review of measurement properties of patient reported outcome measures in psoriatic arthritis: A GRAPPA-OMERACT initiative. *Seminars in Arthritis and Rheumatism*. 10.1016/j.semarthrit.2017.09.002 29037523

[21] Leung, Y. Y., Png, M. E., Wee, H. L., & Thumboo, J. (2013). Comparison of EuroQol-5D and short form-6D utility scores in multiethnic Asian patients with psoriatic arthritis: A cross-sectional study. *Journal of Rheumatology*. 10.3899/jrheum.120782 23504382

[22] Brodszky, V., Péntek, M., Bálint, P. V., Géher, P., Hajdu, O., Hodinka, L., & Gulácsi, L. (2010). Comparison of the Psoriatic Arthritis Quality of Life (PsAQoL) questionnaire, the functional status (HAQ) and utility (EQ-5D) measures in psoriatic arthritis: results from a cross-sectional survey. *Scandinavian Journal of Rheumatology*. 10.3109/03009740903468982 20166848

[23] Prinsen, C. A. C., Mokkink, L. B., Bouter, L. M., Alonso, J., Patrick, D. L., de Vet, H. C. W., & Terwee, C. B. (2018). COSMIN guideline for systematic reviews of patient-reported outcome measures. *Quality of life research : An international journal of quality of life aspects of treatment, care and rehabilitation,**27*(5), 1147–1157. 10.1007/s11136-018-1798-3 29435801 PMC5891568

[24] Mokkink, L. B., Prinsen, C. A., Patrick, D. L., Alonso, J., Bouter, L. M., de Vet, H. C., & Terwee, C. B. (2019). COSMIN Study Design checklist for Patient-reported outcome measurement instruments.

[25] Terwee, C. B., Bot, S. D., de Boer, M. R., van der Windt, D. A., Knol, D. L., Dekker, J., … de Vet, H. C. (2007). Quality criteria were proposed for measurement properties of health status questionnaires. *J Clin Epidemiol*. 10.1016/j.jclinepi.2006.03.012 17161752

[26] Dolan, P. (1997). Modeling valuations for EuroQol health states. *Medical Care,**35*(11), 1095–1108. 10.1097/00005650-199711000-00002 9366889

[27] Burström, K., Sun, S., Gerdtham, U.-G., Henriksson, M., Johannesson, M., Levin, L. -Å., & Zethraeus, N. (2014). Swedish experience-based value sets for EQ-5D health states. *Quality of Life Research,**23*(2), 431–442. 10.1007/s11136-013-0496-4 23975375 PMC3967073

[28] Prevoo, M. L., van ’t Hof, M. A., Kuper, H. H., van Leeuwen, M. A., van de Putte, L. B., & van Riel, P. L. (1995). Modified disease activity scores that include twenty-eight-joint counts. Development and validation in a prospective longitudinal study of patients with rheumatoid arthritis. *Arthritis and rheumatism*, *38*(1), 44–48. 10.1002/art.1780380107 7818570

[29] Schoels, M., Aletaha, D., Funovits, J., Kavanaugh, A., Baker, D., & Smolen, J. S. (2010). Application of the DAREA/DAPSA score for assessment of disease activity in psoriatic arthritis. *Annals of the Rheumatic Diseases*. 10.1136/ard.2009.122259 20525844

[30] Nell-Duxneuner, V. P., Stamm, T. A., Machold, K. P., Pflugbeil, S., Aletaha, D., & Smolen, J. S. (2010). Evaluation of the appropriateness of composite disease activity measures for assessment of psoriatic arthritis. *Annals of the Rheumatic Diseases*. 10.1136/ard.2009.117945 19762363

[31] Salaffi, F., Ciapetti, A., Carotti, M., Gasparini, S., & Gutierrez, M. (2014). Disease activity in psoriatic arthritis: Comparison of the discriminative capacity and construct validity of six composite indices in a real world. *BioMed Research International*. 10.1155/2014/528105 24967375 PMC4055291

[32] Fransen, J., Antoni, C., Mease, P. J., Uter, W., Kavanaugh, A., Kalden, J. R., & Van Riel, P. L. (2006). Performance of response criteria for assessing peripheral arthritis in patients with psoriatic arthritis: Analysis of data from randomised controlled trials of two tumour necrosis factor inhibitors. *Annals of the Rheumatic Diseases*. 10.1136/ard.2006.051706 16644783 PMC1798317

[33] Aletaha, D., Alasti, F., & Smolen, J. S. (2017). Disease activity states of the DAPSA, a psoriatic arthritis specific instrument, are valid against functional status and structural progression. *Annals of the Rheumatic Diseases*. 10.1136/annrheumdis-2016-209511 27457512

[34] Pardo, E., Charca, L., Alonso, S., Alperi, M., Arboleya, L., & Queiro, R. (2020). Disease Activity in Psoriatic Arthritis Index and Psoriatic Arthritis Impact of Disease Questionnaire: correlation and sensitivity to change in a real clinical setting. *Clin Exp Rheumatol*. 31969229

[35] Antoni, C. E., Kavanaugh, A., Kirkham, B., Tutuncu, Z., Burmester, G. R., Schneider, U., Furst, D. E., Molitor, J., Keystone, E., Gladman, D., Manger, B., Wassenberg, S., Weier, R., Wallace, D. J., Weisman, M. H., Kalden, J. R., & Smolen, J. (2005). Sustained benefits of infliximab therapy for dermatologic and articular manifestations of psoriatic arthritis: Results from the infliximab multinational psoriatic arthritis controlled trial (IMPACT). *Arthritis and Rheumatism*. 10.1002/art.20967 15818699

[36] Fransen, J., & van Riel, P. L. C. M. (2009). The Disease Activity Score and the EULAR response criteria. *Rheumatic diseases clinics of North America*, *35*(4), 745–757, vii–viii. 10.1016/j.rdc.2009.10.001 19962619

[37] Schoels, M. M., Aletaha, D., Alasti, F., & Smolen, J. S. (2016). Disease activity in psoriatic arthritis (PsA): Defining remission and treatment success using the DAPSA score. *Annals of the Rheumatic Diseases*. 10.1136/annrheumdis-2015-207507 26269398

[38] Fries, J. F., Spitz, P., Kraines, R. G., & Holman, H. R. (1980). Measurement of patient outcome in arthritis. *Arthritis and Rheumatism,**23*(2), 137–145. 10.1002/art.1780230202 7362664

[39] Wan, M. T., Walsh, J. A., Craig, E. T., Husni, M. E., Scher, J. U., Reddy, S. M., & Ogdie, A. (2021). A comparison of physical function instruments in psoriatic arthritis: HAQ-DI vs MDHAQ vs PROMIS10 global physical health. *Rheumatology (Oxford)*. 10.1093/rheumatology/keaa591 33313838 PMC8599834

[40] Svensk Reumatologis Kvalitetsregister. (2023). BESÖKSBLANKETT - Patient. Retrieved July 31, 2025, from https://srq.nu/wp-content/uploads/2023/10/Besoksblankett-alla-diagnoser.pdf

[41] Hewlett, S., Hehir, M., & Kirwan, J. R. (2007). Measuring fatigue in rheumatoid arthritis: A systematic review of scales in use. *Arthritis and rheumatism,**57*(3), 429–439. 10.1002/art.22611 17394228

[42] Lati, C., Guthrie, L. C., & Ward, M. M. (2010). Comparison of the construct validity and sensitivity to change of the visual analog scale and a modified rating scale as measures of patient global assessment in rheumatoid arthritis. *The Journal of rheumatology,**37*(4), 717–722. 10.3899/jrheum.090764 20194445 PMC2959173

[43] Sendlbeck, M., Araujo, E. G., Schett, G., & Englbrecht, M. (2015). Psychometric properties of three single-item pain scales in patients with rheumatoid arthritis seen during routine clinical care: A comparative perspective on construct validity, reproducibility and internal responsiveness. *RMD Open,**1*(1), Article e000140. 10.1136/rmdopen-2015-000140 26719815 PMC4692050

[44] Tamiya, N., Araki, S., Ohi, G., Inagaki, K., Urano, N., Hirano, W., & Daltroy, L. H. (2002). Assessment of pain, depression, and anxiety by visual analogue scale in Japanese women with rheumatoid arthritis. *Scandinavian journal of caring sciences,**16*(2), 137–141. 10.1046/j.1471-6712.2002.00067.x 12000666

[45] Veehof, M. M., ten Klooster, P. M., Taal, E., van Riel, P. L. C. M., & van de Laar, M. A. F. J. (2008). Comparison of internal and external responsiveness of the generic Medical Outcome Study Short Form-36 (SF-36) with disease-specific measures in rheumatoid arthritis. *The Journal of rheumatology,**35*(4), 610–617. 18322989

[46] Aletaha, D., & Smolen, J. S. (2007). The Simplified Disease Activity Index (SDAI) and Clinical Disease Activity Index (CDAI) to monitor patients in standard clinical care. *Best practice & research. Clinical rheumatology,**21*(4), 663–675. 10.1016/j.berh.2007.02.004 17678828

[47] Bruce, B., & Fries, J. F. (2003). The Stanford Health Assessment Questionnaire: Dimensions and practical applications. *Health and quality of life outcomes,**1*, 20. 10.1186/1477-7525-1-20 12831398 PMC165587

[48] Thyberg, I., Dahlström, Ö., Björk, M., Arvidsson, P., & Thyberg, M. (2012). Potential of the HAQ score as clinical indicator suggesting comprehensive multidisciplinary assessments: The Swedish TIRA cohort 8 years after diagnosis of RA. *Clinical rheumatology,**31*(5), 775–783. 10.1007/s10067-012-1937-0 22249375

[49] Kapetanovic, M. (2024). Psoriasisartrit. Retrieved July 31, 2025, from https://www.internetmedicin.se/reumatologi/psoriasisartrit

[50] Fritz, C. O., Morris, P. E., & Richler, J. J. (2012). Effect size estimates: Current use, calculations, and interpretation. *Journal of experimental psychology. General,**141*(1), 2–18. 10.1037/a0024338 21823805

[51] Coolican, H. (2014). *Research methods and statistics in psychology* (6th ed). Psychology Press.

[52] Marra, C. A., Woolcott, J. C., Kopec, J. A., Shojania, K., Offer, R., Brazier, J. E., Esdaile, J. M., & Anis, A. H. (2005). A comparison of generic, indirect utility measures (the HUI2, HUI3, SF-6D, and the EQ-5D) and disease-specific instruments (the RAQoL and the HAQ) in rheumatoid arthritis. *Social Science & Medicine,**60*(7), 1571–1582. 10.1016/j.socscimed.2004.08.034 15652688

[53] Al Sayah, F., Jin, X., Short, H., McClure, N. S., Ohinmaa, A., & Johnson, J. A. (2025). A systematic literature review of important and meaningful differences in the EQ-5D index and visual analog scale scores. *Value in Health,**28*(3), 470–476. 10.1016/j.jval.2024.11.006 39694263

[54] Gülfe, A., Geborek, P., & Saxne, T. (2005). Response criteria for rheumatoid arthritis in clinical practice: How useful are they? *Annals of the rheumatic diseases,**64*(8), 1186–1189. 10.1136/ard.2004.027649 15760931 PMC1755621

[55] Degerlund-Maldi, K., Regardt, M., Nystrand Länsman, C., Larsson, L., Parodis, I., & Heintz, E. (2026). The value of EQ-5D-3L and EQ VAS as a patient-reported outcome measure for patients with ankylosing spondylitis in routine healthcare: An evaluation of construct validity and responsiveness based on the Swedish Rheumatology Quality Register. *Journal of Patient-Reported Outcomes*. 10.1186/s41687-026-01009-0 41653349 PMC13057080

[56] Herdman, M., Gudex, C., Lloyd, A., Janssen, M. F., Kind, P., Parkin, D., Bonsel, G., & Badia, X. (2011). Development and preliminary testing of the new five-level version of EQ-5D (EQ-5D-5L). *Quality of Life Research,**20*(10), 1727–1736. 10.1007/s11136-011-9903-x 21479777 PMC3220807

[57] Devlin, N. J., Xie, F., Slaap, B., & Stolk, E. (2025). Measuring and valuing health using EuroQol instruments: New developments 2025 and beyond. *Applied Health Economics and Health Policy,**23*(6), 947–960. 10.1007/s40258-025-00989-2 40705271 PMC12535937

[58] Dewilde, S., Janssen, M. F., Phillips, G., Mulhern, B., & Rencz, F. (2026). Comparing psychometric properties of 6 of the 5-level and 3-level EQ-5D bolt-ons in a large, multinational, longitudinal general population sample. *Value in Health*. 10.1016/j.jval.2025.09.009 41005701

[59] Teni, F. S., Burström, K., Devlin, N., Parkin, D., & Rolfson, O. (2023). Experience-based health state valuation using the EQ VAS: A register-based study of the EQ-5D-3L among nine patient groups in Sweden. *Health and Quality of Life Outcomes,**21*, Article 34. 10.1186/s12955-023-02115-z 37038172 PMC10084671

[60] Svensk Reumatologis Kvalitetsregister. (2026, March 5). Täckningsgrad i SRQ för SpA och PsA. *Svensk Reumatologis Kvalitetsregister*. Retrieved March 26, 2026, from https://srq.nu/tackningsgrad-i-srq-for-spa-och-psa/

