# Supplementary material for: Construct validity and responsiveness of EQ-5D-3L and EQ VAS in psoriatic arthritis: an evaluation based on the Swedish Rheumatology Quality Register
Source: Qual Life Res. 2026 Jul 23;35(9):231. doi: 10.1007/s11136-026-04323-8 (PMC13395909; doi:10.1007/s11136-026-04323-8)
Supplement: Supplementary file 1 — Supplementary file1 (DOCX 181 KB) [file 11136_2026_4323_MOESM1_ESM.docx]

Article title: Construct Validity and Responsiveness of EQ-5D-3L and EQ VAS in Psoriatic Arthritis: An Evaluation based on the Swedish Rheumatology Quality Register

Journal name: Quality of Life Research

Author names: Kinza Degerlund-Maldi (corresponding author), Malin Regardt, Camilla Nystrand Länsman, Lena Larsson, Ioannis Parodis, Emelie Heintz.

Affiliation and e-mail for corresponding author: Health Economic and Policy Research Group, Department of Learning, Informatics, Management and Ethics (LIME), Karolinska Institutet, 171 77 Stockholm, Sweden.
Stockholm Center for Health Economics, Center for Health Economics, Informatics and Health Services Research (CHIS), Stockholm Healthcare Services, 171 77 Stockholm, Sweden.
kinza.degerlund.maldi.2@ki.se

# Supplementary material

## Visual analogue scales (VAS)

Authors' translation of the three VAS included in the study.

Question:
“How much pain have you experienced over the past week due to your rheumatic disease?”

Scale where the patient indicates their answer:
No pain --------------------------------------------------------------------------------------------- Worst pain imaginable

Question:
“How fatigued have you been over the past week due to your rheumatic disease?”

Scale where the patient indicates their answer:
No fatigue -------------------------------------------------------------------------------------- Worst fatigue imaginable

Question:
”How have you generally felt over the past week, considering your rheumatic disease?”

Scale where the patient indicates their answer:
Completely well ----------------------------------------------------------------------------------- As bad as imaginable

## Hypothesised correlations

Table S1. Hypotheses on the correlations between EQ-5D-3L (index and descriptive system), EQ VAS, and comparator instruments for assessing convergent validity and responsiveness

|  |  | EQ-5D-3L dimension | | | | |  |
| --- | --- | --- | --- | --- | --- | --- | --- |
| Comparator instruments | EQ-5D-3L index | Mobility | Self-care | Daily activities | Pain/  discomfort | Anxiety/  depression | EQ VAS |
| DAS28 CRP | -- | ++ | ++ | ++ | ++ |  | -- |
| DAPSA | -- | ++ | ++ | ++ | ++ |  | -- |
| HAQ-DI | -- | ++ | ++ | ++ | ++ |  | -- |
| VAS pain | -- | ++ | ++ | ++ | +++ |  | -- |
| VAS fatigue | -- | ++ | ++ | ++ |  | ++ | -- |
| VAS general health | --- |  |  |  |  |  | --- |

The plus and minuses represent the direction and the strength of the correlation. ++: at least a moderate ≥0.3 positive correlation (related constructs), +++: a large ≥0.5 positive correlation (similar constructs). Negative symbols follow the same strength interpretation but indicate negative correlations. For responsiveness changes in scores of the instruments were correlated.
DAPSA, Disease Activity Index in Psoriatic Arthritis; DAS28 CRP, Disease Activity Score 28 CRP; HAQ-DI, Health Assessment Questionnaire Disability Index; VAS, Visual Analogue Scale.

## Sensitivity analyses

Table S2. Correlations between the EQ-5D-3L index (Swedish experience-based value set) and comparator instruments assessing convergent validity and responsiveness

| Convergent validity | | Responsiveness | |
| --- | --- | --- | --- |
| Comparator instruments | EQ-5D-3L index | Comparator instruments | EQ-5D-3L index |
| **DAS28 CRP**  n = 12,868 | **-0.58** | **DAS28 CRP**  n = 2,213 | **-0.43** |
| **DAPSA**  n = 8,472 | **-0.67** | **DAPSA**  n = 1,280 | **-0.48** |
| **HAQ-DI**  n = 13,016 | **-0.74** | **HAQ-DI**  n = 2,216 | **-0.52** |
| **VAS pain**  n = 13,100 | **-0.69** | **VAS pain**  n = 2,257 | **-0.53** |
| **VAS fatigue**  n = 12,795 | **-0.65** | **VAS fatigue**  n = 1,793 | **-0.40** |
| **VAS general health**  n = 13,105 | **-0.70** | **VAS general health**  n = 2,264 | **-0.51** |

Bold text with green shading indicates that the hypothesis was supported. All correlations are significant at the 0.01 level (2-tailed). DAPSA, Disease Activity Index in Psoriatic Arthritis; DAS28 CRP, Disease Activity Score 28 CRP; HAQ-DI, Health Assessment Questionnaire Disability Index; VAS, Visual Analogue Scale.

Table S3. Known-groups validity: Mean EQ-5D-3L index with the Swedish experience-based value set

|  | DAS28 CRP | |  | DAPSA | |  | HAQ-DI | |  |
| --- | --- | --- | --- | --- | --- | --- | --- | --- | --- |
|  | <3.2  n = 9,016 | ≥3.2  n = 3,852 |  | ≤14  n = 5,447 | >14  n = 3,025 |  | <1  n = 9,022 | ≥1  n = 3,994 |  |
| Mean  EQ-5D-3L index | 0.856 | 0.728 | p < 0.001  ES 1.06^a^  **0.128^b^** | 0.876 | 0.724 | p < 0.001  1.33^a^  **0.152^b^** | 0.874 | 0.690 | p < 0.001  ES 1.70^a^  **0.184^b^** |

Bold text with green shading indicates that the hypothesis was supported. DAPSA, Disease Activity Index in Psoriatic Arthritis; DAS28 CRP, Disease Activity Score 28 CRP; ES, effect size; HAQ-DI, Health Assessment Questionnaire Disability Index; p, p-value.
^a^ Effect size calculated with Cohen’s d, ^b^ Difference in mean EQ-5D-3L index value between the groups

Table S4. Area under the receiver operating characteristic curve for the EQ-5D-3L (Swedish experience-based value set)

|  | AUC (95% CI) |
| --- | --- |
| **DAS28 CRP** n = 2,213 | **0.713 (0.692-0.735)** |
| **DAPSA** n = 1,280 | **0.724 (0.696-0.751)** |
| **HAQ-DI** n = 2,216 | **0.751 (0.730-0.772)** |

Bold text with green shading indicates that the hypothesis was supported. AUC, Area Under the Curve; CI, Confidence Interval; DAPSA, Disease Activity Index in Psoriatic Arthritis; DAS28 CRP, Disease Activity Score 28 CRP; HAQ-DI, Health Assessment Questionnaire Disability Index.

Table S5. Responsiveness: correlations between changes in EQ-5D-3L and comparator instruments, restricted to patients with a recorded date of diagnosis

|  |  | EQ-5D-3L descriptive system | | | | |  |
| --- | --- | --- | --- | --- | --- | --- | --- |
| Comparator instruments | **EQ-5D-3L Index**  n = 1,308 | **Mobility**  n = 1,308 | **Self-care**  n = 1,308 | **Usual activities**  n = 1,308 | **Pain/**  **discomfort**  n = 1,308 | **Anxiety/**  **depression**  n = 1,308 | **EQ VAS**  n = 263 |
| **DAS28 CRP**  n = 1,276 | **-0.41** | 0.25 | 0.19 | 0.22 | **0.30** | (0.15) | **-0.42** |
| **DAPSA**  n = 851 | **-0.49** | 0.25 | 0.19 | 0.25 | **0.36** | (0.17) | **-0.38** |
| **HAQ-DI**  n = 1,293 | **-0.52** | **0.33** | 0.27 | **0.33** | **0.34** | (0.20) | **-0.40** |
| **VAS pain**  n = 1,292 | **-0.55** | **0.31** | 0.21 | 0.28 | 0.38 | (0.22) | **-0.45** |
| **VAS fatigue**  n = 1,268 | **-0.36** | 0.18 | 0.13 | 0.20 | (0.22) | 0.23 | **-0.41** |
| **VAS general health**  n = 1,308 | **-0.51** | (0.26) | (0.19) | (0.24) | (0.34) | (0.27) | **-0.50** |

Bold text with green shading indicates that the hypothesis was supported, while regular text with yellow shading indicates that the hypothesis was not supported. Correlations shown in brackets represent cases where no hypothesis was specified. Spearman’s Rho was used for the EQ-5D-3L index value and the EQ VAS, and Kendall’s Tau was used for the descriptive system. All correlations are significant at the 0.01 level (2-tailed).
DAPSA, Disease Activity Index in Psoriatic Arthritis; DAS28 CRP, Disease Activity Score 28 CRP; HAQ-DI, Health Assessment Questionnaire Disability Index; VAS, Visual Analogue Scale.

Table S6. Area under the receiver operating characteristic curve of EQ-5D-3L index value and EQ VAS including only patients with a date of diagnosis.

|  | EQ-5D-3L |  | EQ VAS |
| --- | --- | --- | --- |
|  | AUC (95% CI) |  | AUC (95% CI) |
| **DAS28 CRP**  n = 1,276 | 0.695 (0.667-0.724) | **DAS28 CRP**  n = 258 | **0.714 (0.651-0.777)** |
| **DAPSA**  n = 851 | **0.705 (0.671-739)** | **DAPSA**  n = 177 | 0.680 (0.602-0.758) |
| **HAQ-DI**  n = 1,293 | **0.751 (0.725-0.778)** | **HAQ-DI**  n = 262 | **0.711 (0.648-0.775)** |

Bold font and green cell indicate that the hypothesis was supported. Regular font and yellow cell indicate that the hypothesis was not supported. AUC, Area Under the Curve; CI, Confidence Interval; DAPSA, Disease Activity Index in Psoriatic Arthritis; DAS28 CRP, Disease Activity Score 28 CRP; HAQ-DI, Health Assessment Questionnaire Disability Index.


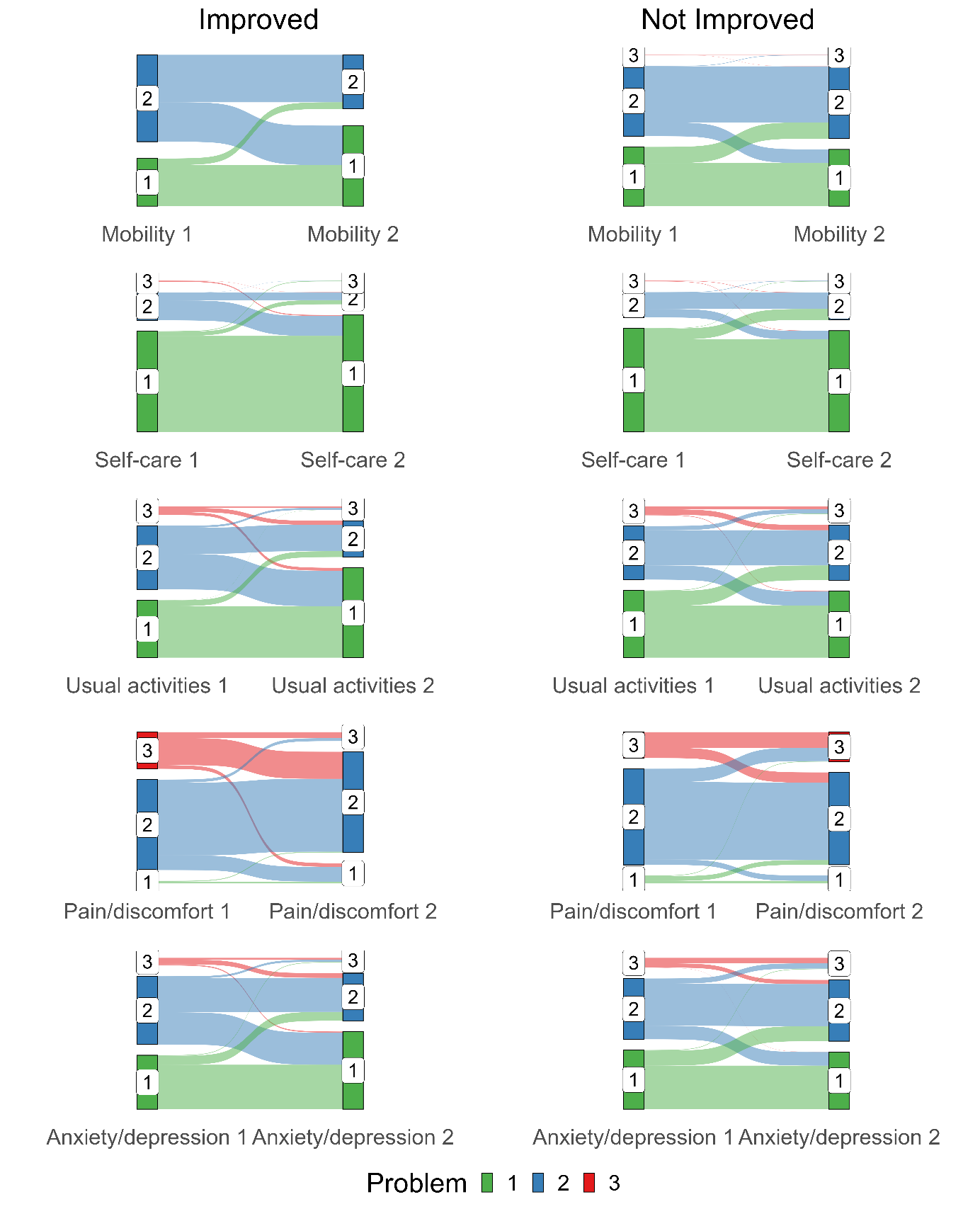


Figure S1. Sankey diagrams illustrating the transition of patient responses across EQ-5D-3L dimensions between the first and second visit stratified by improvement in DAS28 CRP. Each pair of plots compares transitions for patients who improved in DAS28 CRP (left column) versus those who did not (right column). 1 = no problem, 2 = some problems, and 3 = extreme problems.
